# Rapid Determination of Nutritional Parameters of Pasta/Sauce Blends by Handheld Near-Infrared Spectroscopy

**DOI:** 10.3390/molecules24112029

**Published:** 2019-05-28

**Authors:** Marina D. G. Neves, Ronei J. Poppi, Heinz W. Siesler

**Affiliations:** 1 Institute of Chemistry, University of Campinas, Campinas CP 6154, Brazil; marina.de.gea.n@gmail.com; 2 Department of Physical Chemistry, University of Duisburg-Essen, D 45117 Essen, Germany; hw.siesler@uni-due.de

**Keywords:** handheld near-infrared spectroscopy, pasta/sauce blends, partial least squares calibration, nutritional parameters

## Abstract

Nowadays, near infrared (NIR) spectroscopy has experienced a rapid progress in miniaturization (instruments < 100 g are presently available), and the price for handheld systems has reached the < $500 level for high lot sizes. Thus, the stage is set for NIR spectroscopy to become the technique of choice for food and beverage testing, not only in industry but also as a consumer application. However, contrary to the (in our opinion) exaggerated claims of some direct-to-consumer companies regarding the performance of their “food scanners” with “cloud evaluation of big data”, the present publication will demonstrate realistic analytical data derived from the development of partial least squares (PLS) calibration models for six different nutritional parameters (energy, protein, fat, carbohydrates, sugar, and fiber) based on the NIR spectra of a broad range of different pasta/sauce blends recorded with a handheld instrument. The prediction performance of the PLS calibration models for the individual parameters was double-checked by cross-validation (CV) and test-set validation. The results obtained suggest that in the near future consumers will be able to predict the nutritional parameters of their meals by using handheld NIR spectroscopy under every-day life conditions.

## 1. Introduction

The miniaturization of vibrational spectrometers has started more than two decades ago, but only within the last decade have real hand-held Raman, MIR (mid-infrared) and near infrared (NIR) scanning spectrometers become commercially available and been utilized for a broad range of analytical applications [1,2,3,4,5,6]. While the weight of the majority of Raman and MIR spectrometers is still in the 1 kg range, the miniaturization of NIR spectrometers has advanced down to the < 100 g level, and developments are under way to integrate them into mobile phones [7,8]. Furthermore, most of the Raman and MIR handheld spectrometers are still in the price range of several ten thousand US$, whereas miniaturized NIR systems have reached the < 500 US$ level. In view of the high price level of Raman and MIR instruments in the near future, only the acquisition of NIR systems can be taken into consideration for private use, whereas handheld Raman and MIR spectrometers will be restricted to industrial, military and homeland security applications, as well as public use, by first responders, customs or environmental institutions.

Because vibrational spectroscopy is a non-invasive technique that allows a rapid and non- destructive analysis [9,10], its use is increasing in analytical applications of food science [11]. In recent years, primarily handheld near-infrared spectroscopy has demonstrated an immense potential in this respect for different purposes such as authentication [12,13,14], classification [15,16,17], quality control [18,19,20,21], the detection of adulteration [22,23,24], and the determination of food parameters [25] such as the preliminary investigations of pasta/sauce mixtures [26,27].

Over the last years public health awareness has grown strongly, and the control of nutritional parameters of everyday life food is just one aspect of this issue. Beyond body weight control, nutritional parameters are directly related to quality of life and disease control, such as as obesity, high cholesterol, gastritis, diabetes and high blood pressure. Thus, in the present study the quantitative analysis of nutritional parameters by handheld NIR spectroscopy is exemplarily demonstrated in detail for different pasta/sauce blends in combination with a chemometric data evaluation. The objective of these investigations is to prove how feasible it will be for consumers in the near future to be able to predict the nutritional parameters of their meals by using handheld NIR spectroscopy [8].

## 2. Experimental Section

### 2.1. Experimental Set-Up

**Figure 1:**
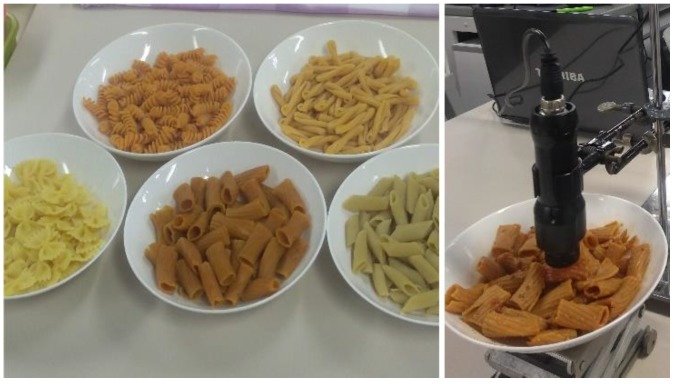
Different morphologies of the investigated pastas and a typical experimental set-up for the measurement of a pasta (here without sauce) with the handheld NIR spectrometer.

For each pasta/sauce-type blend five different combinations (ranging from a 0% to 100% (*w*/*w*) sauce addition) were investigated. Each pasta/sauce mixture was prepared “ready-to-eat” on a plate, and the NIR spectra were recorded at room temperature (22 ± 1 °C) at a distance of 1–2 mm above the sample surface at five different positions of the plate in order to compensate inevitable compositional and surface heterogeneities (Figure 1). Previous investigations have shown that the effective pathlength of NIR radiation for diffuse reflection measurements varies (wavelength and material dependent) from several hundred micrometers to millimeters [28,29,30].

### 2.2. Instrumentation

Near-infrared spectra were measured in diffuse reflection with a Viavi MicroNIR 1700 (formerly JDSU, Santa Rosa, CA, USA) handheld spectrometer, based on a linear variable filter (LVF) monochromator.

The five replicate spectra were recorded with an integration time of 8.8 ms by averaging 1000 scans in the wavelength range of 908–1676 nm with an uncooled 128 pixel InGaAs array detector at a spectral resolution of 12.5 nm at 1000 nm. The S/N ratio derived from the 100% line, recorded with the parameters given above, was 5067:1. As reference, a 99% Spectralon reflectance standard (Labsphere Inc., North Sutton, NH, USA) was used.

### 2.3. Materials

Five different commercial pastas (Farfalle—Edeka, Italy; Tortiglioni—Birkel, Germany; Penne—GutBio, Germany; Fusilli de lentilles corail—Barilla, Italy; Casarecce de pois chiches—Barilla, Italy) and five different commercial tomato sauces (Ricotta—Barilla, Italy; Gorgonzola—Barilla, Italy; Zucchini & Aubergine—Barilla, Italy; Siciliana—Bertolli, Italy; Kräuter—Knorr, Germany) were used for the preparation of the samples. Both the pastas and the sauces were carefully selected to represent a large variation of nutritional parameters and morphologies, in order to develop representative chemometric PLS [31,32] models for the individual parameters of energy, fat, protein, carbohydrates, sugar and fiber. The nutritional parameter values of the calibration mixtures were calculated from the package labels of the pastas and sauces according to the mixture compositions and are summarized in Table 1. The mutual assignment of the five sauces to the five pastas established 25 basic combinations, and for each combination five different proportions of pasta and sauce were prepared by mixing 75 g of dry pasta with five different weights of sauce (0.00 g, 18.75 g, 37.50 g, 56.25 g and 75.00 g). These proportions correspond to pasta/sauce blend ratios (%(*w*/*w*)) of 100/0, 100/25, 100/50, 100/75, and 100/100. Before mixing, the dry pastas were cooked by boiling in water for 10 min, and after draining for a defined time period of 5 min they were put on the plate, and the sauces were added and mixed with the pastas. Thus, 125 plates in total were prepared, and five replicate spectra were measured for each plate, yielding 625 NIR spectra for further processing and analysis. 

### 2.4. Spectral Preprocessing Treatment

**Figure 2:**
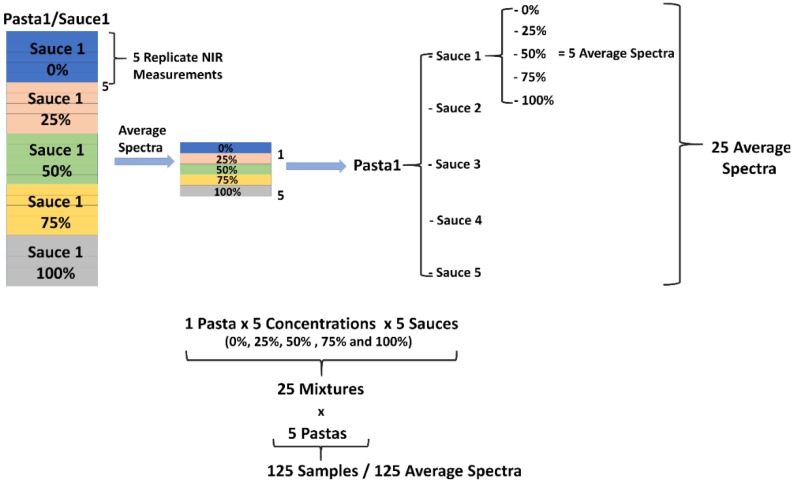
Sample preparation and spectra acquisition scheme demonstrated exemplarily for Pasta 1 and Sauce 1.

**Figure 3:**
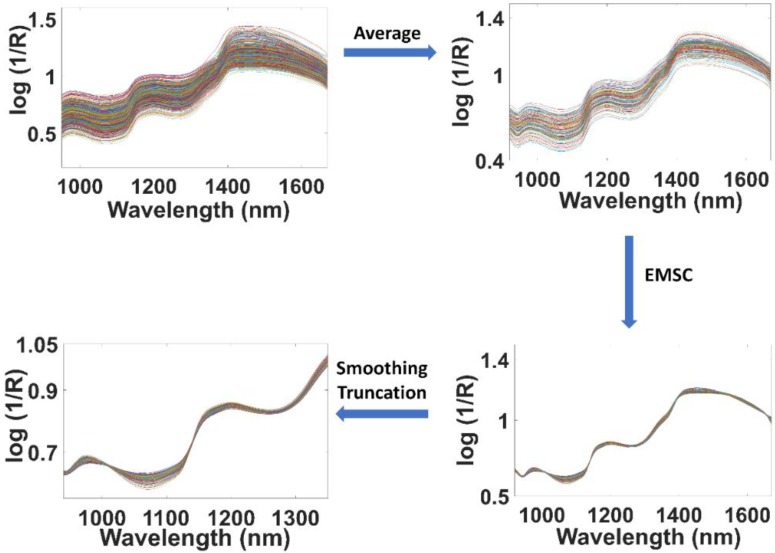
Pretreatments applied to the NIR spectra recorded for the pasta/sauce mixtures.

In Figure 2, the sample preparation and spectra acquisition scheme is exemplarily demonstrated with specific reference to the pasta-1/sauce-1 blends. Thus, in a first step, the average spectra of the replicate measurements were calculated, and the resulting 125 spectral datasets were then concatenated in a matrix. In the matrix containing the average spectra, Savitzky-Golay (SG) smoothing [33] was applied by using a window-size 7 and 2nd degree polynomial, followed by an extended multiplicative scatter correction (EMSC) [34,35,36]. Finally, the spectral range was truncated to 950–1350 nm. The effects of the subsequent pretreatment steps on the original 625 raw spectra are demonstrated in detail in Figure 3.

### 2.5. Chemometric Data Analysis 

Individual PLS calibrations with mean centering and leave-one-out cross validation (CV) were developed for the different nutritional parameters with MatLab software (version R2016a, The MathWorks, Inc., Natick, MA, USA) and the PLS toolbox (version 8.6., Eigenvector Inc., Manson, WA, USA). 

For the separation of the available pasta/sauce mixtures into calibration and test samples for the different nutritional parameters, the 125 samples were arranged by increasing order of the respective parameter, and one sample was removed randomly from each consecutive group of five samples. The 100 remaining samples were used as the calibration set, whereas the 25 removed samples were used as the test set. The test set samples were finally used for an additional validation step and the demonstration of the predictive capability for “unknown” samples.

## 3. Results and Discussion

**Figure 4:**
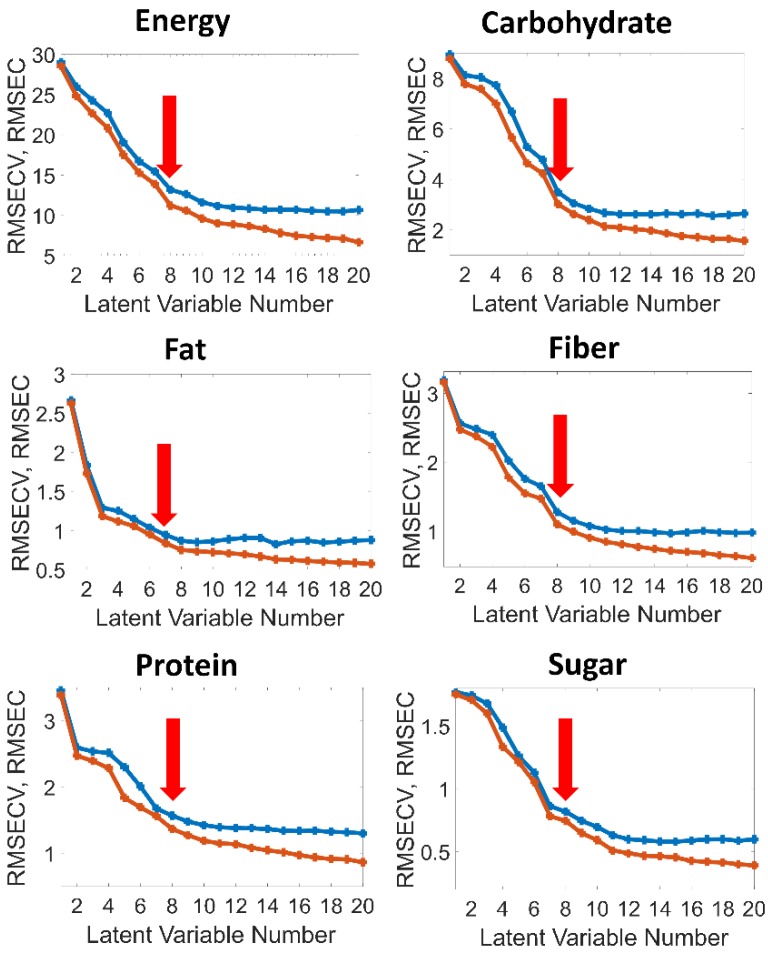
RMSEC (red) and RMSECV (blue) versus the latent variable number for the individual calibrations of the nutritional parameters.

The choice of the number of latent variables (factors) is a critical point in the PLS model development and should be based on the relation to other statistical parameters such as RMSEC and RMSECV [37]. Figure 4 shows plots of the RMSEC/RMSECV values versus the latent variable number for the individual calibrations of the nutritional parameters. Basically, the selection is a compromise between the magnitude of error, robustness of calibration and overfitting. In the present case, eight factors were chosen for energy, carbohydrate, sugar, fiber and protein, respectively, and only seven factors for fat, because the graphs of the RMSEs versus the number of latent variables flatten out beyond these numbers of latent variables (red arrows in Figure 4).

**Figure 5:**
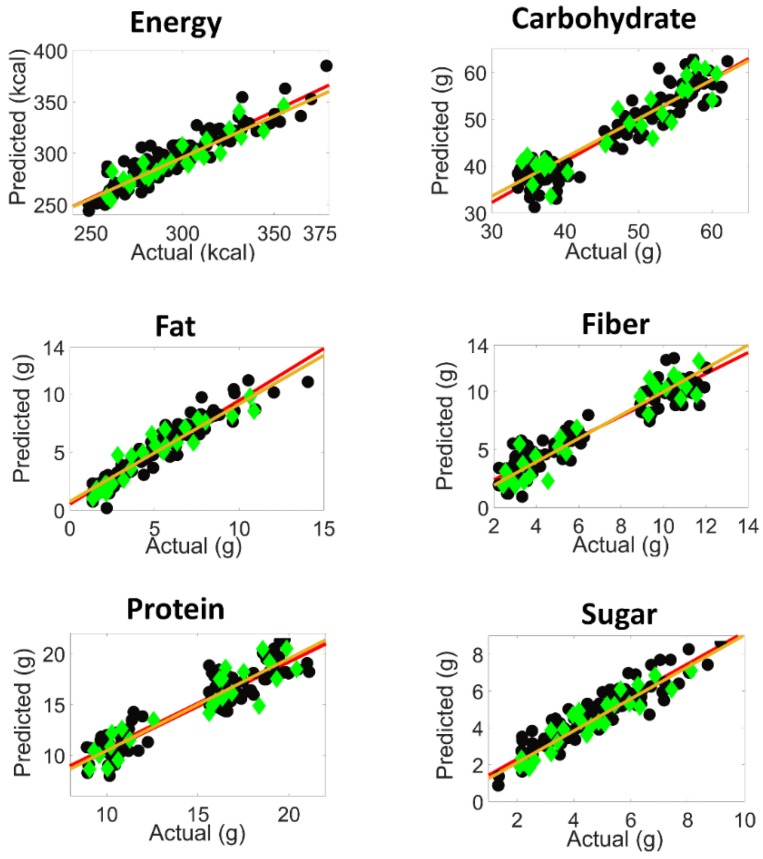
Graphs of the predicted versus actual content of the respective nutritional parameter per serving (calibration fit (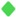), prediction fit (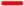), calibration samples (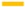) and predicted test set samples (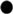)).

The comparatively high number of factors can be readily explained by the complexity of the samples under investigation. Apart from the fact that six parameters are determined, the samples were prepared with five different types of pastas with varying morphologies and sauces, with considerable variations of ingredients (vegetables, cheese, etc.). Furthermore, residual amounts of water lead to hydrogen bonding interactions with carbohydrates, sugars, fibers, and proteins. In Table 2, the content ranges and selected calibration parameters such as root mean square error of calibration (RMSEC), root mean square error of cross validation (RMSECV), root mean square error of prediction (RMSEP), bias, slope, offset and correlation, have been summarized. The residual predictive deviation (RPD) was also included to estimate how well the calibration model can predict the compositional data [37,38]. Generally, the RMSEs and RPDs shown in Table 2 furnish evidence that, at best, medium quality calibrations have been achieved that can be used for the screening purposes of the nutritional parameters under investigation. In Figure 5, the predicted versus actual concentration graphs are shown for the calibration and test set samples for all nutritional parameters, with a linear regression fit. As an additional feature, this figure also reflects two classes of calibration samples for the parameters of carbohydrate, protein and fiber. 

An overview of the prediction results for the test set samples is provided in Table 3 and Table 4. The predictions for energy and carbohydrate of the test set samples were obtained with R^2^Cal 0.85 and 0.89, respectively, and average relative prediction errors of 2.7 and 6.4 %(*w*/*w*), respectively. Protein had an R^2^Cal of 0.87 and an average relative prediction error of 8.3 %(*w*/*w*). The calibrations for sugar and fat led to R^2^Cal values of 0.86 and 0.91, respectively, and average relative prediction errors of 11.4 and 16.1 %(*w*/*w*), respectively. With an R^2^Cal of 0.89, the largest average relative prediction error of 18.2 %(*w*/*w*) was obtained for the fiber calibration model. The comparatively large relative prediction errors for fat, sugar and fiber are not really unexpected and are partly due to the much lower content of these components and, for sugar and fiber, they are a consequence of the structural similarity with the main component carbohydrate. A comparison of the regression vectors of carbohydrate and sugar (not shown here), for example, highlighted an almost identical pattern of important wavelength variables for their calibration models. However, although the NIR spectra contain overlapping features, the PLS method takes into account both the spectral information and the reference nutritional values when building the quantification models. Thus, despite the addressed structural similarity, it is still possible to reasonably quantify the sugar and fiber parameters, as shown in Table 2, Table 3 and Table 4.

## 4. Conclusions

In combination with chemometric evaluation routines, NIR spectroscopy has proved a powerful analytical tool for authentication, adulteration and quality control in food science. The presented method, using a miniaturized spectrometer and PLS calibration models to quantify nutritional parameters of pasta/sauce mixtures, is simple, fast and non-destructive. The achieved calibration results provide an overview of the realistically expectable prediction accuracy for quantifying energy, carbohydrate, fat, fiber, protein and sugar via the application of handheld instruments. However, the results also demonstrate that the “cloud-derived” concentration data reported by several direct-to-consumer companies in commercial videos and advertising papers are beyond any realistic accuracy that is achievable with their relatively simple food-scanners.

## Figures and Tables

**Table 1:** Nutritional parameter values calculated for 100 g of dry pasta and 100 g of sauce.

Sample	Energy (kcal)	Carbohydrate (g)	Fat (g)	Fiber (g)	Protein (g)	Sugar (g)
**Pasta**						
**1**	374.0	75.0	1.8	3.0	13.5	3.0
**2**	347.0	69.0	2.1	4.0	12.0	6.0
**3**	360.0	61.0	2.8	6.5	21.0	3.4
**4**	335.0	47.4	2.9	12.0	25.0	1.8
**5**	348.0	45.1	7.3	14.0	21.0	2.9
**Sauce**						
**1**	97.0	6.8	7.7	1.8	3.4	5.0
**2**	136.0	8.6	11.3	2.0	3.0	6.5
**3**	74.0	6.6	4.7	2.1	1.5	4.8
**4**	91.0	6.0	7.4	1.4	1.5	5.4
**5**	33.0	4.8	0.9	1.0	1.0	3.9

**Table 2:** Content Range and statistical parameters obtained for the individual PLS models of the nutritional parameters.

Parameter	Energy	Carbohydrate	Fat	Fiber	Protein	Sugar
**# LVs**	8	8	7	8	8	8
**RMSEC**	11.15 ^a^	2.97 ^b^	0.83 ^b^	1.10 ^b^	1.36 ^b^	0.65 ^b^
**RMSECV**	13.10 ^a^	3.43 ^b^	0.94 ^b^	1.27 ^b^	1.56 ^b^	0.74 ^b^
**RMESEP**	10.64 ^a^	3.59 ^b^	0.95 ^b^	1.11 ^b^	1.39 ^b^	0.61 ^b^
**Content Range**	248.67–378.54 ^a^	33.55–62.13 ^b^	1.34–14.06 ^b^	2.23–12.03 ^b^	8.89–21.67 ^b^	1.34–9.15 ^b^
**R^2^ Cal**	0.85	0.89	0.91	0.89	0.87	0.86
**R^2^ CV**	0.80	0.85	0.88	0.85	0.83	0.82
**R^2^ Pred**	0.86	0.85	0.89	0.90	0.86	0.88
**RPD**	2.02	2.54	2.77	2.45	2.26	2.19
**Slope CV**	0.85	0.89	0.91	0.89	0.87	0.86
**Offset CV**	43.12 ^a^	5.21 ^b^	0.46 ^b^	0.73 ^b^	1.92 ^b^	0.62 ^b^
**Slope Pred**	0.80	0.83	0.84	0.99	0.91	0.87
**Offset Pred**	55.0 ^a^	8.81 ^b^	0.75 ^b^	0.37 ^b^	1.40 ^b^	0.38 ^b^

^a^ = kcal; ^b^ = g.

**Table 3:** The actual and predicted nutritional parameter content and relative error obtained for the test set samples per serving via the individual PLS models developed for energy, carbohydrates and fat.

Energy (kcal)			Carbohydrate (g)			Fat (g)		
Actual	Predicted	Relative Error (%)	Actual	Predicted	Relative Error (%)	Actual	Predicted	Relative Error (%)
299.8	307.9	2.7	60.1	54.1	9.9	1.4	1.0	25.3
355.2	346.3	2.5	59.1	61.1	3.4	6.8	7.1	4.4
281.4	275.7	2.0	60.7	59.8	1.3	4.8	6.5	35.1
331.9	315.8	4.9	57.8	61.3	6.2	1.6	1.8	16.7
285.7	282.0	1.3	56.0	56.2	0.3	5.7	5.5	2.8
260.8	254.4	2.5	51.7	54.0	4.5	3.2	2.6	17.9
279.3	277.7	0.6	56.6	59.3	4.7	7.3	5.8	20.5
330.9	340.5	2.9	54.5	50.0	8.3	1.7	1.6	9.2
289.4	287.4	0.7	56.7	56.4	0.5	3.6	4.7	27.9
258.6	258.4	0.1	53.3	51.6	3.2	5.1	5.6	10.0
271.2	272.2	0.4	45.5	44.2	3.0	4.3	5.0	15.2
307.5	299.7	2.5	47.2	52.0	10.3	2.1	2.3	8.4
344.2	321.6	6.6	48.8	49.0	0.4	2.2	1.5	32.8
325.6	323.5	0.7	50.4	48.3	4.2	3.6	3.6	1.0
303.1	288.7	4.8	51.9	45.7	12.0	10.6	9.8	8.1
320.6	300.2	6.4	45.7	44.7	2.3	5.6	7.0	23.7
267.7	274.8	2.7	50.1	45.6	8.9	2.2	2.2	1.8
293.6	291.8	0.6	35.5	36.2	2.0	7.6	7.8	3.8
302.3	290.5	3.9	37.2	39.4	5.8	2.5	2.3	9.4
278.5	290.8	4.4	40.3	39.1	3.1	2.8	4.7	67.4
271.2	268.7	0.9	37.9	40.2	6.1	6.3	5.8	8.4
311.6	296.4	4.9	36.1	39.9	10.6	8.0	7.6	5.2
313.0	313.3	0.1	38.4	45.4	18.1	5.5	5.0	8.9
261.4	282.3	8.0	37.2	40.7	9.4	9.6	8.1	15.5
286.0	282.5	1.2	34.1	41.3	21.1	10.9	8.5	21.6
**Average Relative Error (%)**		**2.7**	**Average Relative Error (%)**		**6.4**	**Average Relative Error (%)**		**16.1**

**Table 4:** The actual and predicted nutritional parameter content and relative error obtained for the test set samples per serving via the individual PLS models developed for fiber, protein and sugar.

Fiber (g)			Protein (g)			Sugar (g)		
Actual	Predicted	Relative Error (%)	Actual	Predicted	Relative Error (%)	Actual	Predicted	Relative Error (%)
3.2	5.5	70.2	12.6	13.5	7.1	3.2	2.6	17.9
3.4	3.8	12.3	10.0	8.7	13.4	3.4	3.2	5.6
2.6	3.1	22.2	10.6	9.5	10.3	6.3	5.1	18.6
2.4	1.9	20.7	11.3	11.5	2.3	2.2	2.1	5.6
2.8	2.4	14.8	10.8	12.6	16.4	8.1	7.1	12.4
4.0	4.4	12.0	10.3	12.2	18.6	4.5	3.6	18.6
3.7	2.7	26.2	9.3	10.5	12.5	5.6	6.1	8.1
3.0	1.9	36.7	9.6	10.0	4.4	6.9	6.9	0.1
3.4	2.2	35.0	9.1	8.6	4.7	7.4	6.1	18.2
4.6	2.3	49.7	10.2	10.8	6.0	5.4	5.3	3.0
5.9	6.9	16.4	15.8	14.9	5.4	6.3	6.3	1.5
5.1	6.1	17.9	18.4	14.9	19.1	3.6	3.9	7.9
5.0	5.2	3.9	16.9	16.1	4.4	4.6	4.1	10.9
5.4	4.7	12.9	16.0	15.3	4.2	2.6	2.2	14.3
10.1	10.3	1.4	15.6	14.2	9.3	3.2	3.9	21.0
10.5	11.4	8.8	19.3	17.5	9.4	4.9	4.3	11.2
9.4	11.1	18.8	19.9	20.5	3.2	2.4	1.8	25.3
8.9	9.6	7.5	20.4	18.5	9.5	2.1	1.9	7.9
9.3	8.0	13.6	18.6	20.5	10.3	4.3	4.4	2.9
9.7	10.4	7.2	18.9	19.2	1.2	6.0	5.3	10.6
10.8	11.0	2.2	16.3	15.7	4.2	2.2	2.3	8.7
11.1	10.4	6.0	17.5	18.2	4.0	4.0	4.7	18.8
11.7	12.6	8.1	16.2	17.5	8.1	4.2	4.9	18.0
10.8	9.4	13.4	16.3	17.5	7.4	5.2	5.3	1.8
11.6	9.7	16.2	16.5	18.6	12.7	5.1	4.2	16.8
**Average Relative Error (%)**		**18.2**	**Average Relative Error (%)**		**8.3**	**Average Relative Error (%)**		**11.4**
